# Aberrant ROS Mediate Cell Cycle and Motility in Colorectal Cancer Cells Through an Oncogenic CXCL14 Signaling Pathway

**DOI:** 10.3389/fphar.2021.764015

**Published:** 2021-10-20

**Authors:** Jun Zeng, Mei Li, Jun-Yu Xu, Heng Xiao, Xian Yang, Jiao-Xiu Fan, Kang Wu, Shuang Chen

**Affiliations:** ^1^ Department of Genetics and Cell Biology, College of Life Sciences, Chongqing Normal University, Chongqing, China; ^2^ Department of Hepatobiliary Surgery, the First Affiliated Hospital of Chongqing Medical University, Chongqing, China; ^3^ Shenzhen Luohu People’s Hospital, the Third Affiliated Hospital of Shenzhen University, Shenzhen, China; ^4^ South China Hospital, Shenzhen University, Shenzhen, China; ^5^ Department of Dermatovenereology, the First Affiliated Hospital of Chongqing Medical University, Chongqing, China

**Keywords:** colorectal cancer, ROS, CXCL14, cell cycle, migration

## Abstract

**Background:** Reactive oxygen species (ROS) act as signal mediators to induce tumorigenesis.

**Objective:** This study aims to explore whether chemokine CXCL14 is involved in the proliferation and migration of ROS-induced colorectal cancer (CRC) cells.

**Methods:** The proliferative and migratory capacities of CRC cells treated with or without H_2_O_2_ were measured by various methods, including the CKK-8 assay, colony formation assay, flow cytometry, wounding healing assay, and migration assay.

**Results:** The results revealed that H_2_O_2_ promoted the proliferation and migration of CRC cells by regulating the cell cycle progression and the epithelial to mesenchymal transition (EMT) process. Furthermore, we noted that the expression level of CXCL14 was elevated in both HCT116 cells and SW620 cells treated with H_2_O_2_. An antioxidant N-Acetyl-l-cysteine (NAC) pretreatment could partially suppress the CXCL14 expression in CRC cells treated with H_2_O_2_. Next, we constructed CRC cell lines stably expressing CXCL14 (HCT116/CXCL14 and SW620/CXCL14) and CRC cell lines with empty plasmid vectors (HCT116/Control and SW620/Control) separately. We noted that both H_2_O_2_ treatment and CXCL14 over-expression could up-regulate the expression levels of cell cycle-related and EMT-related proteins. Moreover, the level of phosphorylated ERK (p-ERK) was markedly higher in HCT116/CXCL14 cells when compared with that in HCT116/Control cells. CXCL14-deficiency significantly inhibited the phosphorylation of ERK compared with control (i.e., scrambled shNCs). H_2_O_2_ treatment could partially restore the expression levels of CXCL14 and p-ERK in HCT116/shCXCL14 cells.

**Conclusion:** Our studies thus suggest that aberrant ROS may promote colorectal cancer cell proliferation and migration through an oncogenic CXCL14 signaling pathway.

## Introduction

Colorectal cancer (CRC) is one of the most common fatal malignancies affecting people worldwide (Sung et al., 2021). The incidence of CRC has increased over the years, especially among individuals of age <45 years (Brody, 2015; Siegel et al., 2017). Emerging evidence has suggested CRC cells exhibit elevated reactive oxygen species (ROS) levels than the normal cells (Lei et al., 2011). ROS can regulate the occurrence and development of cancer in an autocrine or paracrine way (Reczek and Chandel, 2018). During intestinal tumorigenesis, myeloid cell-derived ROS triggered oxidative DNA damage in intestinal epithelial cells to stimulate invasive growth in a paracrine manner (Canli et al., 2017). Anti-oxidant treatment can reduce cholangiocellular pre-neoplastic lesions (Yuan et al., 2017). Although accumulating evidence suggests the oncogenic role of ROS, high or excessive levels of ROS can cause damage to the cellular components, leading to cell death (Li et al., 2015; He et al., 2016; Zhuang et al., 2016; Salehi et al., 2018; Ruiz-Torres et al., 2019; Zhang et al., 2019).

Chemokines have been reported to regulate the proliferation and metastasis of tumor cells in an organ-specific manner. CXCL14, a CXC chemokine ligand, chemoattracts proinflammatory cells, such as natural killer (NK) cells and dendritic cells (DCs), to the sites of inflammation or malignancy (Shurin et al., 1950; Starnes et al., 2006). CXCL14 plays a double-sided role in the regulation of tumor development. In head and neck squamous cell carcinoma (HNSCC), CXCL14 could be used as a functional prognosis biomarker for patients’ better overall survival rate (Li et al., 2020a; Wu et al., 2021). On the contrary, CXCL14 was reported to promote cancer cell motility by regulating Ca^2+^ release in breast cancer (Pelicano et al., 2009a). Previously, we reported that chemokine CXCL14 was significantly upregulated in the CRC tissues than that in the normal and paracancerous tissues (Zeng et al., 2013a). Whether the contradictory functions of CXCL14 in tumor progression are due to the types of tumors awaits further investigation.

The relationship between ROS and chemokines during carcinogenesis has been studied (Wen et al., 2018; Li et al., 2020b; Dasoveanu et al., 2020; Xian et al., 2020; Alafiatayo et al., 2020; da Ros et al., 2015). A recent study demonstrated that topoisomerase inhibitors could promote the expression and secretion of CXCL1 *via* ROS-mediated activation of JAK2-STAT1 signaling pathway, thereby promoting the motility of cancer cells (Liu et al., 2019). ROS-mediated CXCL8 expression could regulate the development of *H. Pylori*-associated gastric cancer (Kyung et al., 2019). Stromal cells in tumor microenvironment could also secret ROS-mediated chemokine and act on tumor cells in a paracrine manner (Ramírez-Moreno et al., 2020). Despite these recent advances, the oncogenic signaling transduction pathways targeted by aberrant ROS levels and chemokines remain to be fully understood.

The purpose of this present study aims to clarify the association between ROS and chemokine CXCL14 in CRC progression, and demonstrate the oncogenic function of chemokine CXCL14 in CRC cells. Herein, we reported experimental evidence for ROS-induced cell cycle progression and the epithelial to mesenchymal transition (EMT) process via CXCL14/pERK pathway in colorectal cancer cells.

## Materials and Methods

### Cell Culture and Treatment

Human CRC cell line HCT116 and SW620 cells were kind gifts from Prof. Yunlong Lei (Molecular Medicine and Cancer Research Center, Chongqing Medical University). Cells were maintained in DMEM medium (Hyclone) containing 10% fetal calf serum (Hyclone), penicillin and streptomycin at 37°C in a humidified atmosphere with 5% CO_2_. The cells were treated without or with H_2_O_2_ in media, which were changed daily. Cells were pretreated with antioxidant 5 mM or 10 mM N-acetylcysteine (NAC) for 30 min before addition of H_2_O_2_.

### Plasmid Constructions and Cell Transfection

To clone the CXCL14 cDNA, we isolated total RNAs from the HCT116 cell line using Trizol method. The RNA samples were dissolved with TE buffer, and then quantified by measuring OD value. The extracted RNA samples were further reverse transcribed into cDNA, which would be used as PCR template. The primers used for CXCL14 amplification were synthesized (Invitrogen). The primer sequences for amplification were as follows: cxcl14, forward 5′-GAT​CCC​CGC​CAC​CAT​GTC​CCT​GCT​CCC​ACG​C-3′, reverse 5′-TCG​AGC​TAT​TCT​TCG​TAG​ACC​CTG​CGC-3’; *gapdh*, forward 5′-ACC​TGA​CCT​GCC​GTC​TAG​AA-3′, reverse 5′-TCC​ACC​ACC​CTG​TTG​CTG​TA-3’. For CXCL14 stable expression in HCT116 cells, the recombinant plasmid of EX-CXCL14-LV203 and the control plasmid EX-NEG-LV203 were transfected into 293T cells with lentivirus packaging system, separately. Then, the concentrated virus particles were collected and the HCT116 cells were infected with the viruses in their logarithmic growth phase. The stable transfectants were selected in the presence of puromycin and identified by real-time PCR and western blotting. The primers for real-time PCR were as follows: cxcl14, forward 5′-CTA​CAG​CGA​CGT​GAA​GAA​GC-3′, reverse 5′-TTC​TCG​TTC​CAG​GCG​TTG​TA-3’. The construction of HCT116 cells lacking CXCL14 was also accomplished *via* lentiviral-mediated transduction with a scrambled shNCs or verified CXCL14-specific shRNA sequence encoded in LVRH1GP. A 21-mer shRNA expressing vector targeting CXCL14 (shCXCL14) and its scrambled sequence-expressing vector as a negative control (shNC) were synthesized (Invitrogen). The sequence for CXCL14 shRNA was forward 5′-GAT​CCG​CAC​CAA​GCG​CTT​CAT​CAA​TTC​AAG​AGA​TTG​ATG​AAG​CGC​TTG​GTG​CTT​TTT​TGG-3′, and reverse 5′-AAT​TCC​AAA​AAA​GCA​CCA​AGC​GCT​TCA​TCA​ATC​TCT​TGA​ATT​GAT​GAA​GCG​CTT​GGT​GCG-3’.

### ROS Measurement

Intracellular ROS were detected by staining cells with 5-(and-6)-chloromethyl-2′,7′-dichlorodihydrofluorescein diacetate (CM-H_2_DCF-DA) (Genmed Scientifics Inc., Burlington, MA, United States). Cells after firm adhesion to the wall were treated with H_2_O_2_ for 6 h. After trypsin digestion and centrifugation, supernatant was removed. 10 μmol/L DCFH-DA dye diluted with serum-free medium was added to cells for incubation at 37°C for 20 min. The CM-H_2_DCF-DA signal was then analyzed with the BD Biosciences FACSCalibur Flow Cytometer (Mountain View, CA).

### Cell Proliferation and Colony Formation Assay

Cell proliferation was determined by using the Cell Counting Kit-8 (CCK-8) Assay (Boster). The cancer cells (5000 cells per well) were seeded in the 96-well plates and treated with different concentrations of H_2_O_2_ for 48 h. The cells were incubated with 10 μL of CCK-8 per well and the optical density (OD) value at 450 nm was determined by microplate reader. The experiment was repeated in triplicate. The formula of promotion rate of cell proliferation (P%) is as follows:

$$P\%=\frac{OD\left(sample\right)-OD\left(control\right)}{OD\left(control\right)-OD\left(blank\right)}\times 100\%$$ (1)

For colony formation assay, the cancer cells (1000 cells per well) were resuspended in DMEM supplemented with 10% FBS without or with H_2_O_2_ in 6-well plates. The cultures were maintained for 14 days and stained with 0.1% crystal violet and estimated under the microscope. Each experiment was performed in triplicate.

### Wound Healing and Cell Migration Assay

Confluent HCT116 cells were wounded with a micropipette tip (200 μL) and immediately placed in 1% serum-containing medium supplemented with or without H_2_O_2_. Bright-field images of wounded monolayers were obtained immediately after wounding (0 h) and at various times thereafter as indicated. The extent of wound closure was quantified by obtaining three wound measurements for each of three random fields (× 100) per wound, and all wound conditions were performed in triplicate.

The cell migration assay was performed in chambers with 8-μm-porosity polycarbonate filter membranes according to the manufacturer’s instructions (Becton Dickinson Labware). Cells (1 × 10^5^ cells per well) treated without or with H_2_O_2_ in serum-free medium were added to the upper chamber and incubated for 20 h. The bottom chamber was prepared with 20% FBS as a chemoattractant. Nonmigrating cells on the upper surface of the membrane were removed. Cells that invaded to the lower surface were fixed with 4% paraformaldehyde stained with crystal violet, and photographed under a microscope (Eclipse TS100, Nikon) at × 20 magnification.

### Western Blot Analysis

Both adherent and floating cells were harvested. All cell protein was extracted in RIPA lysis buffer (50 mM Tris, 1.0 mM EDTA, 150 mM NaCl, 0.1% SDS, 1% Triton X-100, 1% sodium deoxycholate, 1 mM PMSF). The concentrations of protein were quantified by using the DC Protein Assay Kit (500–0121; Bio-Rad). The cellular lysates were resolved on SDS-PAGE and electrophoretically transferred to polyvinylidene difluoride membranes. The membranes were then blocked with a buffer [composed of 10 mM Tris (Zhu et al., 2018), 150 mM NaCl, 0.1% Tween 20 and 5% bovine serum albumin], and then incubated with the primary antibodies (CXCL14, diluted 1:400, Proteintech, 10468-1-AP; Cyclin A1, diluted 1:1000–2000, Abcam, ab53699; Cyclin B1, diluted 1:1000–2000, Abcam, ab215436; CDK1, diluted 1:1000–2000, Abcam, ab265590; CDK2, diluted 1:1000–2000, Abcam, ab101682; E-cadherin, diluted 1:1000–2000, Abcam, ab238099; N-cadherin, diluted 1:1000–2000, Abcam, ab207608; vimentin, diluted 1:1000–2000, Abcam, ab92547; ERK, diluted 1:1000–2000, Abcam, ab184699; p-ERK, diluted 1:1000–2000, Abcam, ab214036; and GAPDH, diluted 1:1000–2000, Abcam, ab181602) at 4°C for overnight. Then, the membranes were washed and treated with appropriate secondary antibodies conjugated with horseradish peroxidase for (goat anti-rabbit IgG H&L, Abcam, ab7090; goat anti-mouse IgG H&L, Abcam, ab7068) 2 h. The immunoreactivities were determined by using enhanced chemiluminescence reagents (WBKLS0500; Millipore) (Wang et al., 2011). The GAPDH protein was used as an internal control.

### Statistical Analysis

Statistical values were defined by using an unpaired Student’s *t*-test. Differences among multiple groups were assessed by one-way ANOVA analysis. *p* < 0.05 was considered to be statistically significant.

## Results

### ROS Promoted the Proliferation and Migration of CRC Cells

**FIGURE 1:**
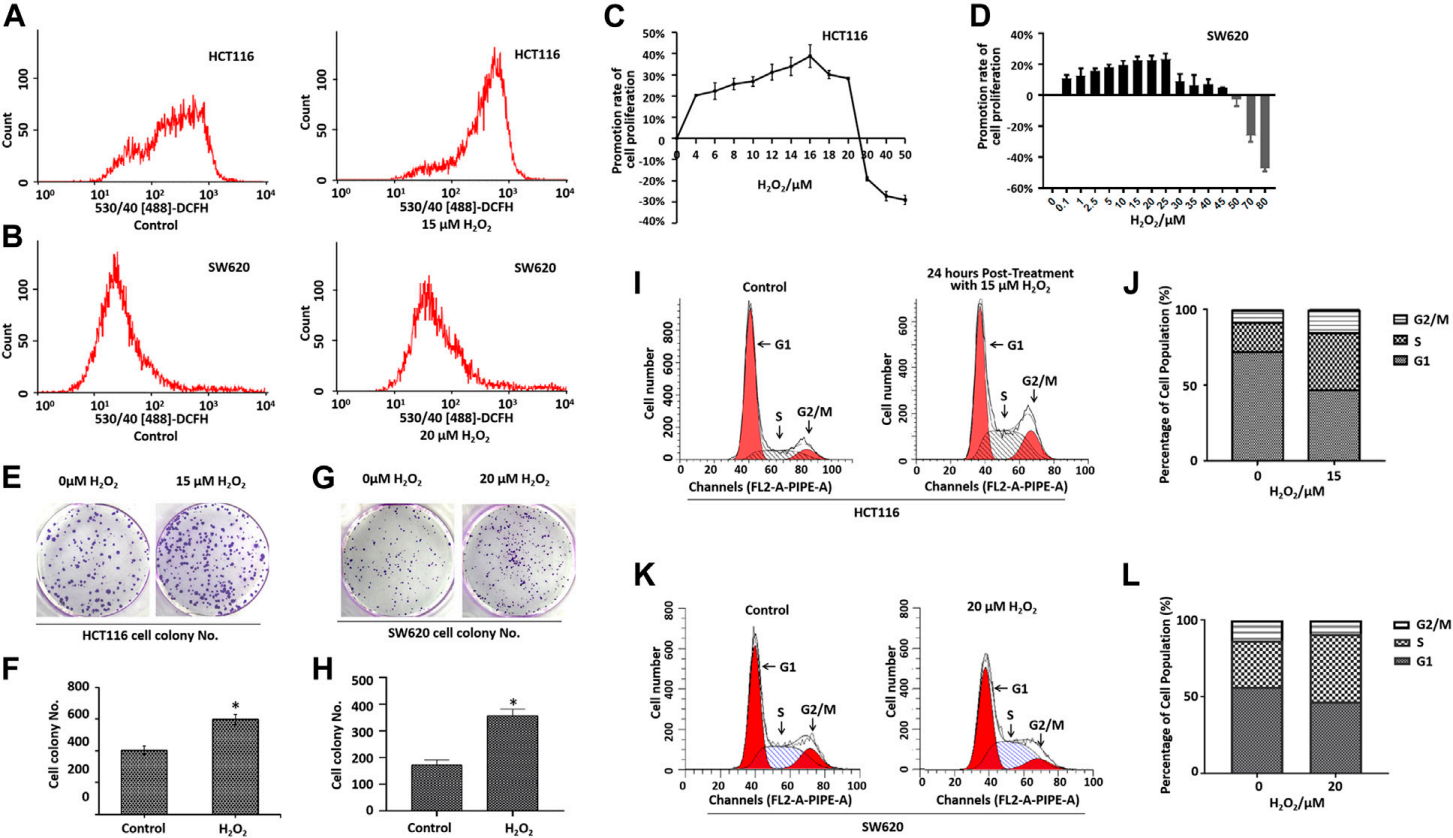
Effects of H_2_O_2_ on colorectal cancer cell growth and cell cycle. Cells treated with the exogenous H_2_O_2_ showed an endogenous increase in the oxidative stress level as measured using CM-H_2_DCF-DA by flow cytometry in HCT116 cells **(A)** and SW620 cells **(B)**. The HCT116 cells **(C)** and SW620 cells **(D)** were treated with different concentrations of H_2_O_2_ for 48 h, and the cell proliferation was determined by CCK8 assay. The calculation of promotion rate of cell proliferation has been described in the Materials and Methods section. Representative photographs of colony formation of HCT116 cells **(E)** and SW620 cells **(G)** treated with or without H_2_O_2_. The statistical analysis of the numbers of cell colonies of HCT116 cells **(F)** and SW620 cells **(H)**. DNA content of HCT116 cells **(I)** and SW620 cells **(K)** treated with or without H2O2 in various phases of the cell cycle measured by flow cytometry. B. Statistical analyses of cell cycle distribution of HCT116 cells **(J)** and SW620 cells **(L)**. The experiments were performed in triplicate (*n* = 3). Error bars represent mean ± S.D. *p* < 0.05 was considered to be statistically significant. *p < 0.05; **p < 0.01.

Recent studies have demonstrated that cancer cells can be characterized by elevated ROS levels when compared with the normal cells (Maurya and Vinayak, 2015; Prasad et al., 2017; Moloney and Cotter, 2018). Aberrant ROS production is associated with tumor initiation and progression (Yang et al., 2016; Ghanbari Movahed et al., 2019). To determine the role of ROS in CRC cell proliferation and migration, we applied exogenous H_2_O_2_ to induce cellular ROS stress in HCT116 cells and SW620 cells. As illustrated in Figures 1A,B, the cells treated with the exogenous H_2_O_2_ showed an endogenous increase in the oxidative stress level when compared with the control cells, as measured by flow cytometry.

Cell proliferation was determined by the CCK-8 assay. As shown in Figures 1C,D, treatment with different doses of H_2_O_2_ induced different biological outcomes. H_2_O_2_ increased HCT116 cell proliferation at a concentration of <25 μM when compared with the control cells. Adversely, H_2_O_2_ decreased the cell proliferation at concentration of >25 μM (Figure 1C). We found a similar phenomenon in SW620 cells (Figure 1D). As expected, the cells treated with H_2_O_2_ formed a greater number of colonies when compared with the control cells (Figures 1E–H). To further characterize the effect of H_2_O_2_ on the cell cycle progression, we examined the DNA content of HCT116 cells and SW620 cells in various phases of the cell cycle, as measured by flow cytometry (Figures 1I–L). Results showed that H_2_O_2_ increased the cell percentage in the S phase and G2/M phase and decreased the cell percentage in the G1 phase in HCT116 cells (Figures 1I,J), and H_2_O_2_ increased the cell percentage in the S phase in SW620 cells (Figures 1K,L).

**FIGURE 2:**
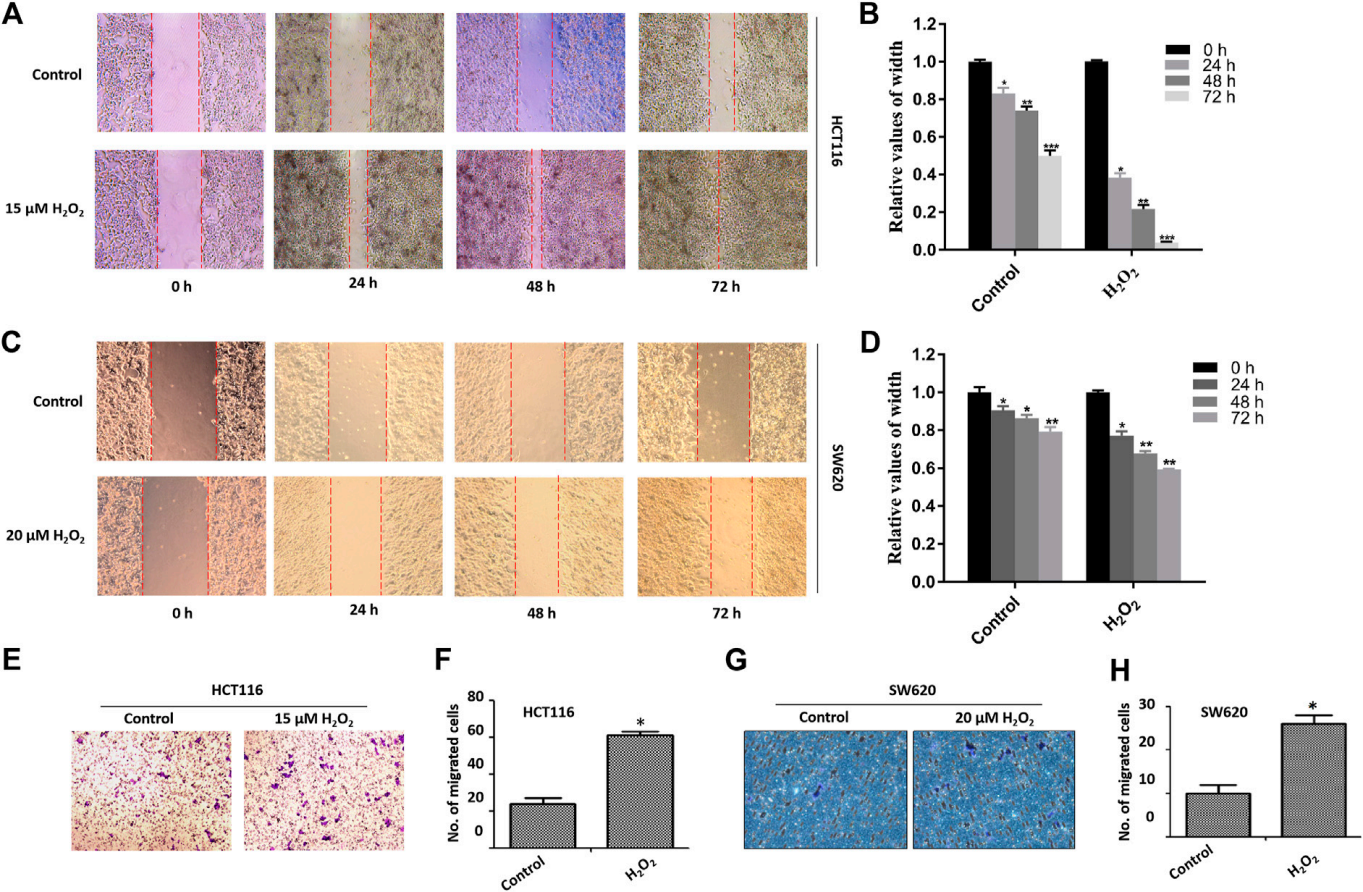
Effects of H_2_O_2_ on CRC cell migration. The HCT116 cells **(A)** and SW620 cells **(C)** were treated with H_2_O_2_ for different time, and the cell migration was determined by the wound healing assay. The width of wound area in HCT116 cells **(B)** and SW620 cells **(D)** was quantitatively analyzed. The migration of HCT116 cells **(E)** and SW620 **(F)** cells treated with or without H_2_O_2_ was measured by the modified Boyden chamber assays. Quantitative results of the numbers of migrated cells in HCT116 cells **(G)** and SW620 cells **(H)**. The experiments were performed in triplicate (*n* = 3). Error bars represent mean ± S.D. *p* < 0.05 was considered to be statistically significant. *p < 0.05; **p < 0.01.

The migratory capacity of HCT116 cells and SW620 cells treated with or without H_2_O_2_ was estimated by the wound healing assay. In the wound healing assay, the number of H_2_O_2_-treated HCT116 and SW620 cells that migrated into the wound area was much greater than the control cells (Figures 2A–D), which suggests the role of ROS in mediating the closure of cell wounds. The cells in the wound area may be a direct reflection of migration, or partially due to cell proliferation. To circumvent this difficulty, we further performed cell migration assays in 24-well transwell chambers. Results showed that ROS significantly increased the migration capacity of the HCT116 cells and SW620 cells (Figures 2E–H). Taken together, these data strongly suggest that ROS is directly involved in mediating the proliferation and migration of CRC cells.

### ROS Promoted Malignant Behaviors in Human CRC Cells *via* Regulation of the Cell Cycle Progression and EMT

**FIGURE 3:**
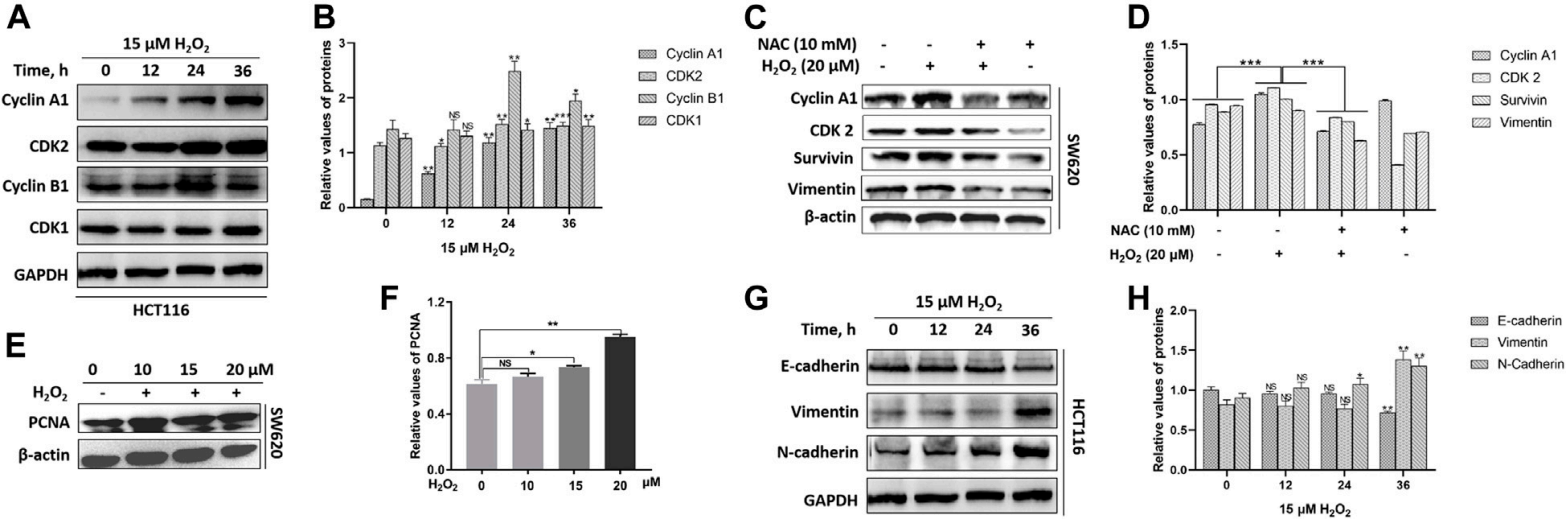
H_2_O_2_ regulated the expression levels of cell cycle-related and EMT-related proteins. **(A)** The HCT116 cells were treated with or without 15 μM H_2_O_2_ for different time. The expression levels of Cyclin A1, CDK2, Cyclin B1, CDK1 were measured by western blot. **(B)** Quantitative results of cell cycle-related protein expression levels. **(C)** The SW620 cells treated with or without 20 μM H_2_O_2_ or pretreated with 10 mM NAC. The expression levels of Cyclin A1, CDK2, surviving and vimentin were measured by western blot, which could be down-regulated by the antioxidant NAC. **(D)** Quantitative results of protein expression. **(E)** The expression of PCNA in SW620 cells treated with different concentration of H_2_O_2_ was measured by western blot. **(F)** Quantitative results of PCNA expression levels. **(G)** The HCT116 cells treated with or without 15 uM H_2_O_2_ for different time. The expression levels of E-cadherin, vimentin and N-cadherin were measured by western blot. **(H)** Quantitative results of EMT-related protein expression levels. All experiments were repeated three times or more. Error bars represent ±S.D. *p* < 0.05 was considered to be statistically significant. * *p* < 0.05; ** *p* < 0.01, *** *p* < 0.001.

In mammalian cells, the cell-cycle progression is highly controlled by cyclins and cyclin-dependent kinases (CDKs). The activity of cyclin A1/CDK2 complex is essential for the cell-cycle G1/S phase transition, and the cyclin B1/CDK1 complex plays the fundamental role in cell-cycle G2/M transition (Malumbres and Barbacid, 2009; Xie et al., 2020). We noted that H_2_O_2_ could up-regulate the expression levels of cyclin A1, cyclin B1, CDK1, and CDK2 in a time-dependent manner in HCT116 cells (Figures 3A,B). H_2_O_2_ could up-regulated the expression of cyclin A1, CDK2 and survivin in SW620 cells, which could be partially suppressed by the antioxidant NAC pretreatment (Figures 3C,D). We also noted that H_2_O_2_ could up-regulate the expression of proliferating cell nuclear antigen (PCNA) in SW620 cells (Figures 3E,F). These data indicated increased growth capacity for the CRC cells in the presence of H_2_O_2_ through regulation of the cell cycle progression and cell proliferation.

The epithelial to mesenchymal transition (EMT) endows cancer cells with the properties of invasion and metastasis (Yuan et al., 2020). In solid tumors, EMT occurs at the invasive front and induces migratory cells with downregulated expression of epithelial markers E-cadherin and upregulated expression of the mesenchymal markers vimentin and N-cadherin (Nieto et al., 2016). By immunoblotting, we observed a substantial decrease in the expression level of E-cadherin and an increase in the expression levels of vimentin and N-cadherin in response to exogenous H_2_O_2_ in HCT116 cells (Figures 3G,H). We also noted that vimentin expression was down-regulated after exposure to H_2_O_2_, which could be partially restored by the antioxidant NAC in SW620 cells (Figures 3C,D).

### ROS Up-Regulated the Expression of Chemokine CXCL14

**FIGURE 4:**
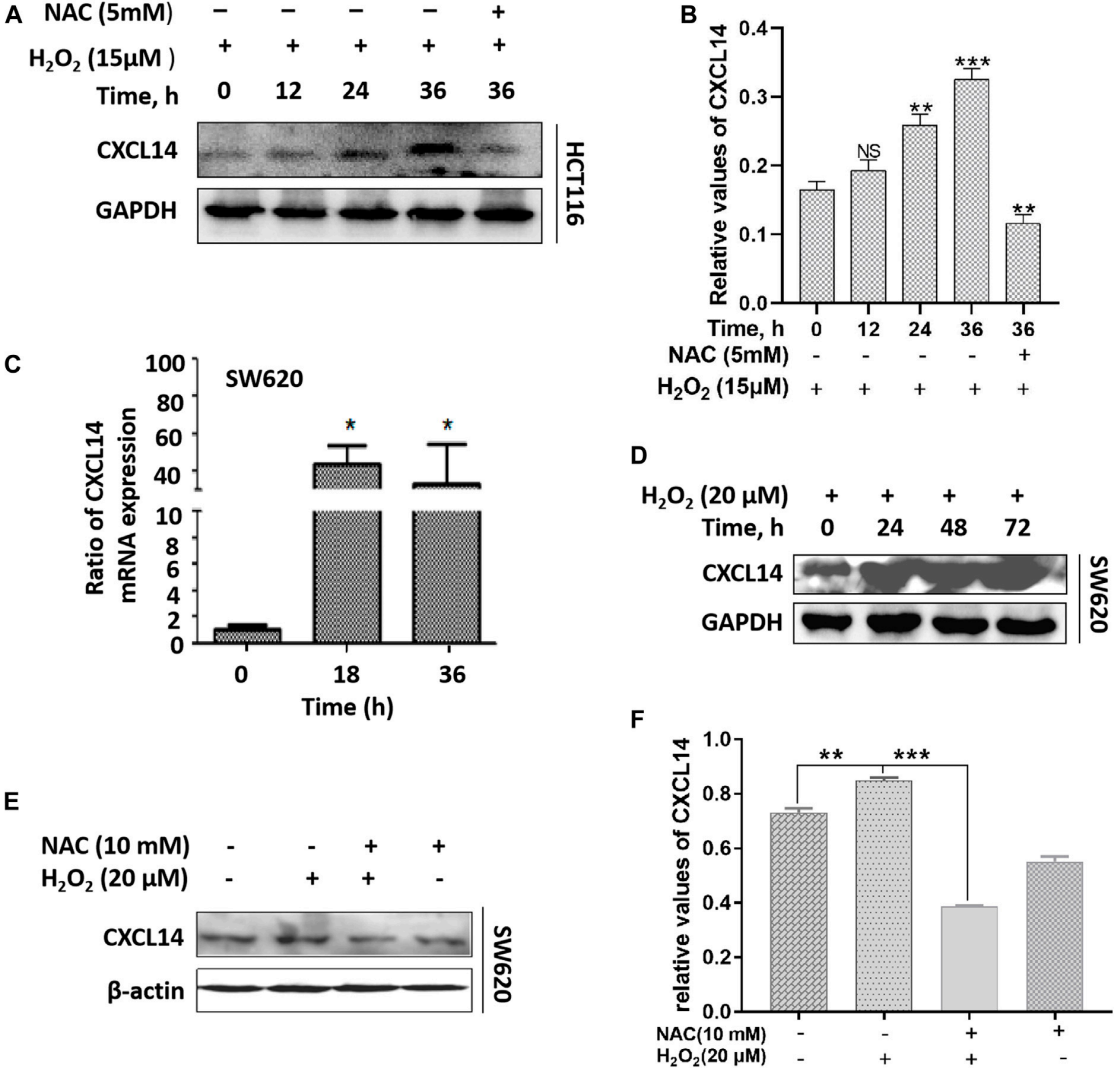
ROS up-regulated the expression of chemokine CXCL14. **(A)** The HCT116 cells were treated with 15 μmol/L H2O2 for different time and/or pretreated with 5 mmol/L NAC. **(B)** Quantitative results of the expression levels of CXCL14. **(C)** The SW620 cells were treated with 20 μmol/L H2O2 for different time. The expression level of CXCL14 mRNA was measured by real-time PCR. **(D)** The SW620 cells were treated with 20 μmol/L H2O2 for different time. The expression level of CXCL14 protein was measured by western blot. **(E)** The SW620 cells were treated with 20 μmol/L H2O2 and/or pretreated with 10 mmol/L NAC. **(F)** Quantitative results of the expression levels of CXCL14. All experiments were repeated three times or more. Error bars represent ± S.D. *p* < 0.05 was considered to be statistically significant. *p <0.05; **p <0.01, ***p <0.001.

Previous studies have shown that ROS can act as signal transduction molecules and regulate the expression of oncogenic chemokines (Wang et al., 2017). Based on previous reports, we performed immunoblotting to examine the expression level of chemokine CXCL14 after exposure to H_2_O_2_ in CRC cells. We observed that the CXCL14 expression was significantly elevated in HCT116 cells treated with H_2_O_2_ when compared with the control cells and in a time-dependent manner (Figures 4A,B). We then used the antioxidant NAC to explore whether the CXCL14 expression was affected. Results showed that NAC pretreatment partially suppressed the expression levels of CXCL14, indicating that CXCL14 expression might be regulated by ROS in HCT116 cells (Figures 4A,B). Meanwhile, we found that the expression levels of CXCL14 mRNA and protein were elevated after exposure of H_2_O_2_ in a time-dependent way in SW620 cells (Figures 4C,D), which could be partially suppressed by the antioxidant NAC pretreatment (Figures 4E,F).

### CXCL14 was Required for Oncogenic Signaling in CRC Cells

**FIGURE 5:**
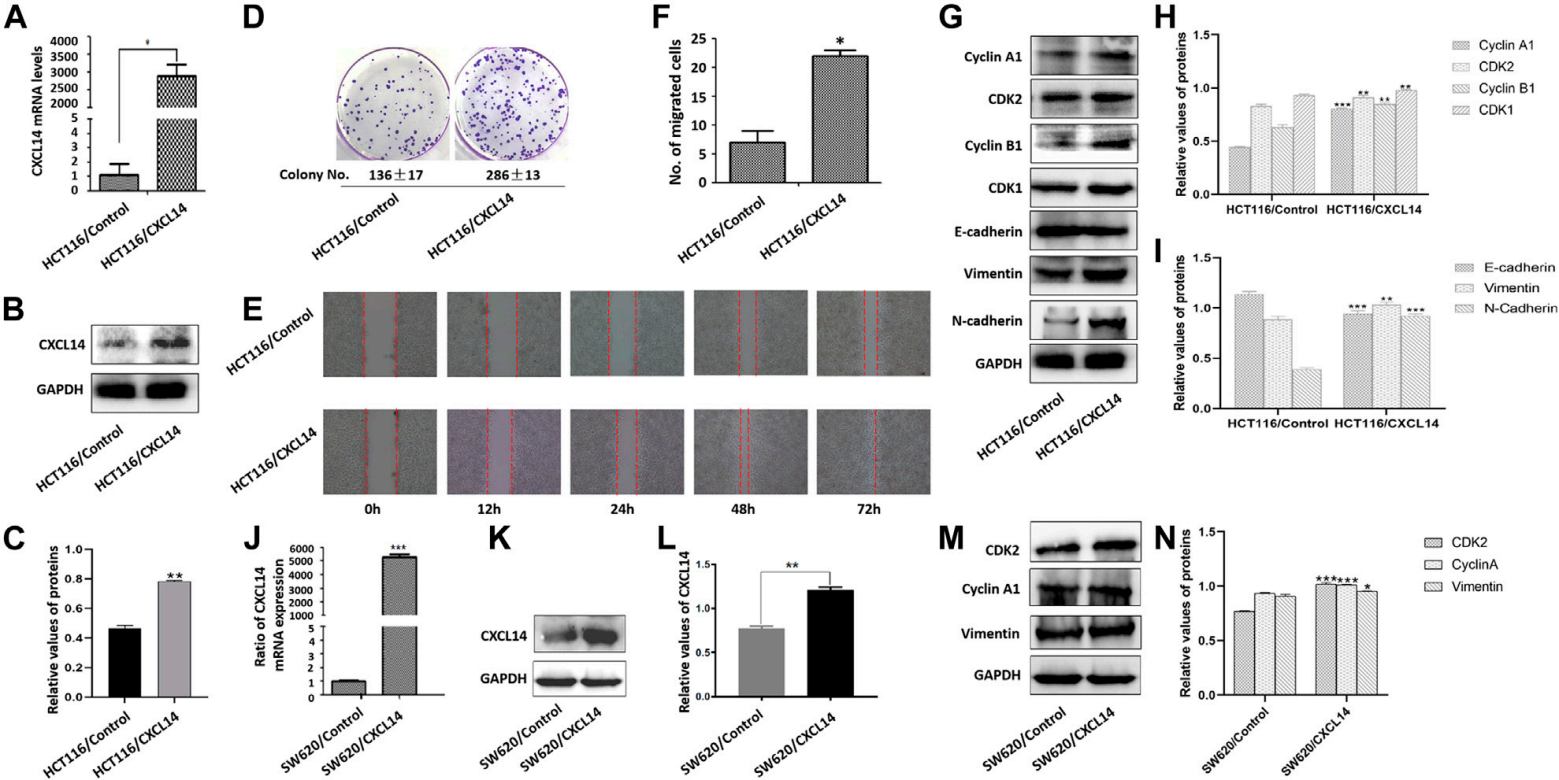
Effects of CXCL14 on cell proliferation and migration of human colorectal cancer cells. CXCL14 was stably over-expressed in the HCT116 cells, as determined by RT-PCR assay **(A)** and western blot assay **(B)**. **(C)** Quantitative results of CXCL14 expression levels. **(D)** The proliferative capacity of HCT116 cells stably expressing CXCL14 (HCT116/CXCL14) was stronger than that of HCT116/Control only transfected with empty vectors, as measured by colony formation assay. **(E)** The migratory capacity of HCT116/CXCL14 was stronger than that of HCT116/control cells, as measured by wound healing assay. **(F)** Quantitative results of cell migration numbers in modified Boyden chamber assays in HCT116/Control cells and HCT116/CXCL14 cells. **(G)** The expression levels of cell cycle-related and EMT-related proteins in HCT116/CXCL14 cells and HCT/Control cells were measured by western blot. **(H)** Quantitative results of cell cycle-related protein expression. **(I)** Quantitative results of EMT-related protein expression. CXCL14 was stably over-expressed in the SW620 cells, as determined by RT-PCR assay **(J)** and western blot assay **(K)**. **(L)** Quantitative results of CXCL14 expression levels. M. The expression levels of CDK2, Cyclin A1, vimentin in HSW620/CXCL14 cells, and SW620/Control cells were measured by western blot. **(H)** Quantitative results of protein expression. The experiments were performed in triplicate (*n* = 3). Error bars represent mean ± S.D. *p* < 0.05 was considered to be statistically significant. * *p* < 0.05; ** *p* < 0.01, *** *p* < 0.001.

To gain further insights into the role of CXCL14 in ROS-induced oncogenesis, we first constructed a stably CXCL14-expressing HCT116 cell line (HCT116/CXCL14), as shown in Figures 5A–C. The proliferative and migratory capacity of HCT116/CXCL14 cells was determined by using the colony formation assay (Figure 5D), wound healing assay (Figure 5E) and migration assay (Figure 5F). As compared with the control cells (HCT116/Control), HCT116/CXCL14 cells exhibited stronger proliferative and migratory capacities, suggesting that CXCL14 might play an important role in CRC progression, which was consistent with previous data (Zeng et al., 2013a).

We further examined the expression levels of cyclin A1, cyclin B1, CDK1, and CDK2, and found that CXCL14 could up-regulate the expression levels of cell cycle-related protein (Figures 5G,H). Meanwhile, we also noted that the HCT116/CXCL14 cells were characterized with downregulation of epithelial markers E-cadherin and upregulation of mesenchymal markers vimentin and N-cadherin (Figures 5G,I), suggesting that CXCL14 may promote the CRC progression through the regulation the EMT process.

We also constructed a stably CXCL14-expressing SW620 cell line (SW620/CXCL14), as shown in Figures 5J–L. We found that the expression levels of CDK2, cyclin B1, and vimentin could be up-regulated by CXCL14 (Figures 5M,N).

Taken together, these data strongly suggests that chemokine CXCL14 may be directly involved in CRC cell proliferation and migration.

### CXCL14 Regulated the ROS-Induced Phosphorylation of ERK

**FIGURE 6:**
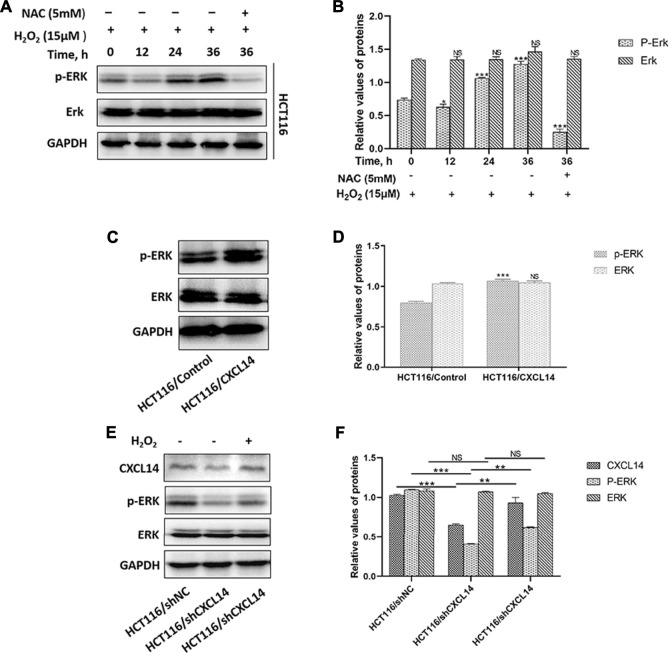
Effects of CXCL14 on the ROS-induced phosphorylation of ERK. **(A)** H_2_O_2_ increased the expression levels of CXCL14 and p-ERK in HCT116 cells treated without or with 15 μM H_2_O_2_ or pretreated with 5 mM NAC in a time-dependent manner. **(B)** Quantitative results of the expression levels of p-ERK. **(C)** CXCL14 could up-regulated the phosphorylation level of ERK in HCT116/CXCL14 cells. **(D)** Quantitative results of the p-ERK and total ERK expression levels in the HCT116/CXCL14 cells and HCT/Control cells. **(E)** CXCL14-deficiency markedly inhibited the phosphorylation of ERK in HCT116/shCXCL14 cells when compared with that in HCT116/shNC. **(F)** Quantitative results of the p-ERK and total ERK expression levels in HCT116/shCXCL14 and HCT116/shNC cells. All experiments were repeated three times or more. Error bars represent ±S.D. *p* < 0.05 was considered to be statistically significant. * *p* < 0.05; ** *p* < 0.01, *** *p* < 0.001.

The typical extracellular-regulated kinase (ERK) cascade is a highly conserved signaling pathway that bridges extracellular signal molecules and intracellular diverse executor proteins, modulating various physiological or pathological processes, including tumor cell proliferation and migration (Maik-Rachline et al., 2019). Aberrant ROS can induce the activation of ERK through different signal cascade reactions in different cancers (Su et al., 2019). In the present study, we found that ROS treatment resulted in a greater accumulation of phosphorylated ERK (p-ERK) in HCT116 cells in a time-dependent manner, which could be partially restored by the antioxidant NAC pretreatment (Figures 6A,B). Previous evidence demonstrated that chemokines could induce cancer development through the activation of Erk1/2 (Zhao et al., 2017). To explore whether chemokine CXCL14 could mediate the level of p-ERK in CRC cells, we compared the levels of p-ERK between HCT116/CXCL14 cells and HCT116/Control cells. The results revealed that the level of p-ERK was markedly higher in the HCT116/CXCL14 cells than that in the HCT116/Control cells (Figures 6C,D). Furthermore, CXCL14-deficiency markedly inhibited the phosphorylation of ERK when compared with the control cells (i.e., scrambled shRNA), as shown in Figures 6E,F. More interestingly, H_2_O_2_ treatment could partially restore the expression levels of CXCL14 and p-ERK in HCT116/shCXCL14 cells (Figures 6E,F).

Taken together, these data suggest that chemokine CXCL14 may stimulate the expression of ROS-induced p-ERK, thereby promoting the CRC progression.

## Discussions

Accumulating evidence suggests that ROS can function as signaling molecules to participate in various physiological and pathological processes, including tumor cell proliferation and motility (Idelchik et al., 2017). The production of ROS can be induced by chronic inflammation, which may further lead to the development of chronic inflammation (Trivedi and Adams, 2018), which is similar to the roles of chemokines in the pathogenesis of chronic inflammation-associated diseases, including CRC (Coussens and Werb, 2002). Based on these backgrounds, we hypothesized that CXCL14 might be involved in ROS-induced CRC progression. To test the hypothesis, we conducted relevant studies and confirmed an important role of CXCL14 in ROS-induced CRC cell proliferation and migration. (a) Exogenous ROS (H_2_O_2_) could promote the proliferation and migration of human CRC cells. (b) ROS could promote the malignant behaviors of human CRC cells by regulating the cell cycle progression and EMT process. (c) CXCL14 was required for oncogenic signaling induced by ROS. (d) ROS-induced CXCL14 stimulated the phosphorylation of ERK in human CRC cells.

In the present study, we demonstrated ROS treatment and CXCL14 overexpression could modulate the expression levels of cell cycle-related proteins (Cyclin A1/B1, CDK1/2) and EMT-related proteins (E-cadherin, N-cadherin, vimentin), suggesting that CXCL14 might be involved in ROS-induced cell cycle progression and EMT process, which was consistent with the role of ROS-induced CXCL14 in breast cancer reported by Pelicano et al. (2009b). However, a previous study showed that ROS stimulated angiogenesis and tumor progression by reducing the expression of CXCL14 *via* EGFR/MEK/ERK signaling pathway in HNSCC cells (Maehata et al., 2010). Oncogenic role of ROS is with no doubt, while CXCL14 may have tumor-suppressive or tumor-supportive functions, depending on the type of the tumor. The conflicting biological functions of CXCL14 in tumor biology have been addressed. CXCL14 plays an anti-tumor role in HNSCC and tongue carcinomaa (Sato et al., 2010; Kondo et al., 2016), but a pro-tumor role in some breast cancer, pancreas cancer, and glioblastoma (Wente et al., 2008; Sjöberg et al., 2016). The contradictory results may be related to the type of the tumor, or dosage and treatment of ROS. CXCL14 may play distinct roles even in the same type of tumor. Gu et al. reported that CXCL14 expression was positively correlated to the overall survival of breast cancer patients as well as lymph node metastasis (Gu et al., 2012). However, Sjoberg et al. reported that high stromal CXCL14 expression correlated with shorter recurrence-free survival of breast cancer patients (Sjöberg et al., 2016). Moreover, CXCL14 expression was reported to be up-regulated by ROS and promoted cell motility in breast cancer cell lines (Pelicano et al., 2009b). In colorectal cancer, there are also some seemingly contradictory studies.

We found that CXCL14 modulated ROS-induced cell proliferation and motility in colorectal cancer cells, suggesting an oncogenic role of CXCL14 in CRC, which was consistent with our previous studies (Zeng et al., 2013b). However, the current study contradicted other findings (Lin et al., 2014; Hata et al., 2015). Lin et al. reported that the expression levels of CXCL14 mRNA and protein in CRC tissues were significantly down-regulated compared with levels in normal tissues (Lin et al., 2014). The clinical sample size and the method of evaluation of immunohistochemical staining may have a certain impact on the statistics and analysis of the results. Sjoberg, et al. divided the expression of CXCL14 in breast cancer clinical samples into three categories: epithelial CXCL14 expression, stromal CXCL14 expression, and total CXCL14 expression. They found that CXCL14 was strongly expressed in stromal cells and stromal CXCL14 expression significantly correlated with shorter survival in breast cancer (Sjöberg et al., 2016). Also, we found that CXCL14 was expressed in stromal cells in CRC specimens (data not shown). If software was adopted to calculate the mean optical density of immunohistochemical staining, the value would include CXCL14 expression in both tumor cells/normal epithelial cells and stromal cells. This evaluation method was different from that we used. Hata et al. reported that the incidence of AOM/DSS-induced cancer was suppressed in the CXCL14 transgenic mice due to the enhanced NK cell activity, implying an anti-tumor role of CXCL14 in chronic colitis-associated carcinogenesis (Hata et al., 2015). Accumulating evidence has demonstrated the multifarious roles of CXCL14 in cancer progression and immune responses. Chemoattraction of iDCs and NK cells and functional maturation of dendritic cells by CXCL14 can substantially contribute to anti-tumor immune response (Shurin et al., 1950; Starnes et al., 2006). In addition to normal epithelial cells, some cancer cells and stromal cells such as cancer-associated fibroblasts (CAFs) in the tumor microenvironment can express and secrete CXCL14 (Sjöberg et al., 2016). In the CXCL14 transgenic mice, all cells could highly express CXCL14, which might affect the anti-tumor or pro-tumor effect of CXCL14 from two aspects. First, whether stable expression of CXCL14 in the CXCL14 transgenic mice affects the function of some immune cells or stromal cells remains to be further explored. Second, there is no significant difference in the expression of CXCL14 between tumor tissues and normal tissues in the CXCL14 transgenic mice. In this way, tumor tissues may have no advantage in chemotacxis of DCs, NK cells or other cells to exert anti-tumor or pro-tumor effects.

Western blotting results revealed that the level of pERK1/2 was markedly higher in HCT116/CXCL14 cells when compared with HCT116/control, and CXCL14-deficiency markedly inhibited the phosphorylation of ERK1/2 compared with control (i.e., scrambled shRNA). Furthermore, ROS treatment could partially restore the expression levels of CXCL14 and pERK1/2 inHCT116/shCXCL14 cells. Thus, CXCL14 seems to provide a potent molecular association between oxidative stress and ERK signaling. In HNSCC cells, the level of phosphorylated ERK was up-regulated after ROS treatment (Maehata et al., 2010), which was consistent with our results. It is the difference that CXCL14 acts as a downstream signal molecule of p-ERK in HNSCC cells and CXCL14 acts as an upstream signal, regulating the phosphorylation level of ERK in CRC cells.

In conclusion, our results established the role of CXCL14 in the ROS-induced CRC cell proliferation and migration to facilitate the development of a rationale for the use of CXCL14 blockers in the treatment and prevention of CRC.

## Data Availability

The data for this study are available by contacting the corresponding author.

## Abbreviations

CAFs, cancer-associated fibroblasts; CCK-8, cell counting kit 8; CDKs, cyclin-dependent kinases; CRC, colorectal cancer; CXCL14, CXC-chemokine ligand 14; DCs, dendritic cells; EMT, epithelial to mesenchymal transition; H2O2, hydrogen peroxide; iDCs, immature dentritic cells; NAC, N-Acetyl-L-cysteine; NK, natural killer; p-ERK, phosphorylated extracellular-regulated kinase; ROS, reactive oxygen species.
